# Obesity: An Immunometabolic Perspective

**DOI:** 10.3389/fendo.2016.00157

**Published:** 2016-12-12

**Authors:** Indrani Ray, Sushil K. Mahata, Rajat K. De

**Affiliations:** ^1^Machine Intelligence Unit, Indian Statistical Institute, Kolkata, India; ^2^Metabolic Physiology & Ultrastructural Biology Laboratory, VA San Diego Healthcare System, La Jolla, CA, USA; ^3^Metabolic Physiology & Ultrastructural Biology Laboratory, University of California San Diego, La Jolla, CA, USA

**Keywords:** obesity, insulin resistance, macrophages, ER stress, reactive oxygen species, type 2 diabetes, non-alcoholic fatty liver diseases

## Abstract

Obesity, characterized by chronic activation of inflammatory pathways, is a critical factor contributing to insulin resistance (IR) and type 2 diabetes (T2D). Free fatty acids (FFAs) are increased in obesity and are implicated as proximate causes of IR and induction of inflammatory signaling in adipose, liver, muscle, and pancreas. Cells of the innate immune system produce cytokines, and other factors that affect insulin signaling and result in the development of IR. In the lean state, adipose tissue is populated by adipose tissue macrophage of the anti-inflammatory M2 type (ATM2) and natural killer (NK) cells; this maintains the insulin-sensitive phenotype because ATM2 cells secrete IL10. In contrast, obesity induces lipolysis and release of pro-inflammatory FFAs and factors, such as chemokine (C–C motif) ligand 2 (CCL2) and tumor necrosis factor alpha (TNF-α), which recruit blood monocytes in adipose tissue, where they are converted to macrophages of the highly pro-inflammatory M1-type (ATM1). Activated ATM1 produce large amounts of pro-inflammatory mediators such as TNF-α, interleukin-1β, IL-6, leukotriene B4, nitric oxide (NO), and resistin that work in a paracrine fashion and cause IR in adipose tissue. In the liver, both pro-inflammatory Kupffer cells (M1-KCs) and recruited hepatic macrophages (Ly6C^high^) contribute to decreased hepatic insulin sensitivity. The present mini-review will update the bidirectional interaction between the immune system and obesity-induced changes in metabolism in adipose tissue and liver and the metabolic consequences thereof.

## Introduction

Multicellular organisms rely on two highly conserved mechanisms for their survival: the ability to store energy to prevent starvation (metabolic pathways) and the ability to fight infection (immune pathways). When nutrients are in excess, adipose tissue stores lipids and the liver stores glycogen for use during starvation or to combat stressful situations. In addition, both adipose tissue and liver are populated with innate and adaptive immune cells. Thus, immune cells modulate whole-body metabolism [in metabolic syndromes such as type 2 diabetes (T2D) and obesity] *via* effects on adipocytes and hepatocytes, and reciprocally, host nutrition and commensal microbiota-derived metabolites modulate immunological homeostasis. This bidirectional interaction between the immune system and whole-body metabolism has created the field of immunometabolism, which has witnessed a renaissance in the past 15 years. The landmark discovery by Hotamisligil et al. in 1993 suggested that tumor necrosis factor (TNF) levels are elevated in the adipose tissue of obese and diabetic rodents and that its neutralization improves insulin-stimulated glucose uptake, which formed the cornerstone for immunometabolism (1). The second groundbreaking discovery in the field of immunometabolism came from Ferrante and Chen’s group, who reported simultaneously that adipose tissue of obese mice is infiltrated with macrophages that contribute to adipose tissue inflammation and IR (2, 3). Since these initial discoveries in immunometabolism, it has been shown that a large number of immune cells and pathways regulate metabolic homeostasis in obese animals (4–11).

Obesity, an epidemic of the twenty-first century, continues to rise throughout the world, even in the countries where poverty and malnutrition are major problems. The World Health Organization estimates that globally there are more than 1.9 billion overweight adults [body mass index (BMI) > 27 kg/m^2^]. Of them, 600 million people are obese with BMI more than 30 kg/m^2^ (WHO obesity and overweight fact sheet, updated in June 2016: http://www.who.int/mediacentre/factsheets/fs311/en/). Obesity provides bacterial and metabolic danger signals that activate a plethora of inflammatory cascades that drives M1 macrophage phenotype. In addition, immune and metabolic pathways are tightly balanced in that the immune response is highly energy demanding and shifts energy away from non-essential functions (12). In contrast, infection and sepsis often result in metabolic disruptions including IR (13). Obesity- and T2D-induced alterations in components of the immune system are most apparent in adipose tissue, the liver, and the pancreatic islets. Therefore, this review will focus on obesity-induced changes in immune system and metabolism in adipose tissue and liver and the consequent development of disease states such as IR, T2D, non-alcoholic fatty liver disease (NAFLD), and non-alcoholic steatohepatitis (NASH).

## Obesity: Innate and Adaptive Immune Responses and Their Signaling

**Figure 1:**
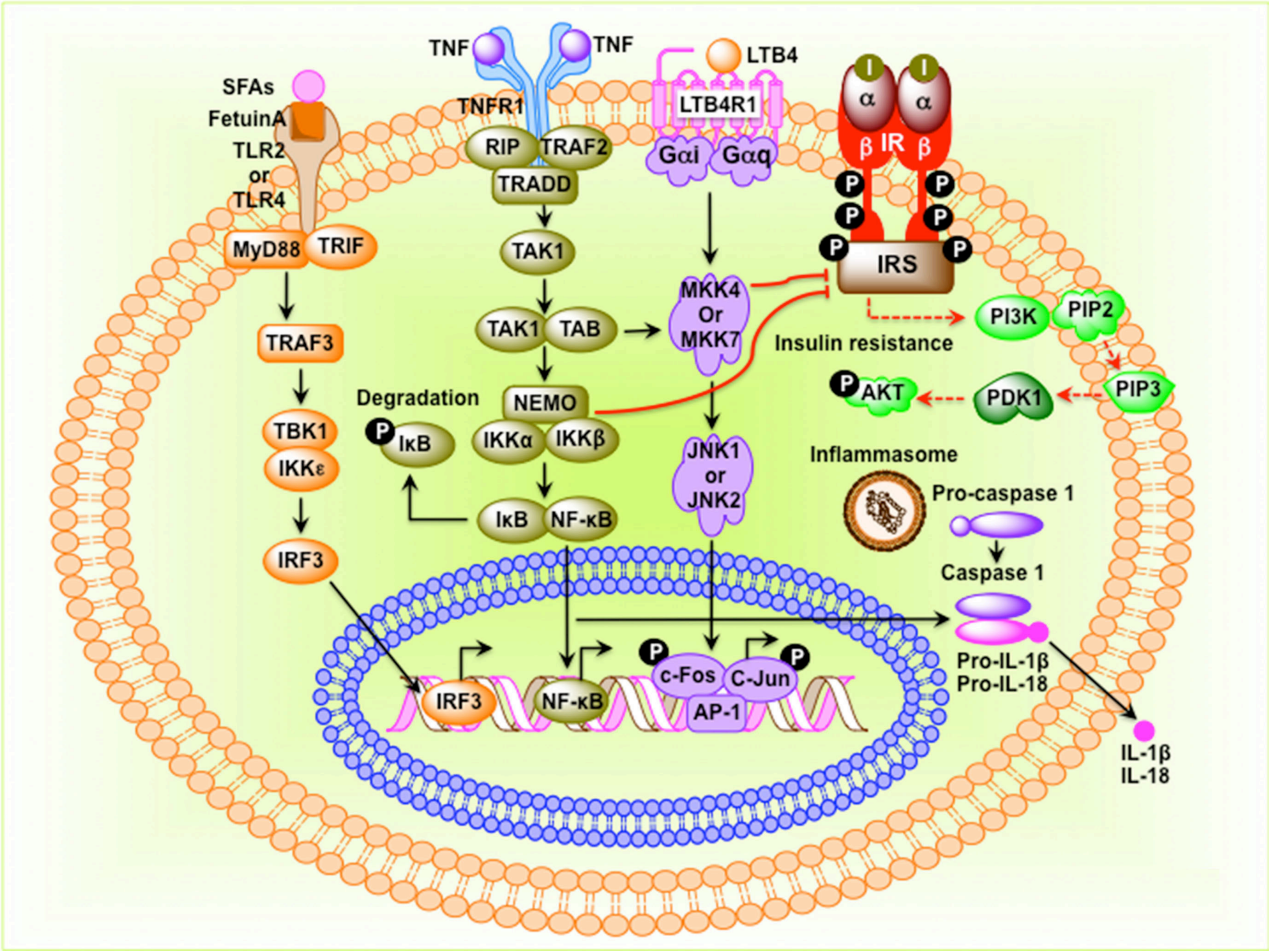
**Molecular events that connect inflammation to insulin resistance in obesity**. Saturated fatty acids (SFAs) bind to Fetuin-A, an endogenous ligand of toll-like receptor 4 (TLR4) and TLR2, and initiate transcription of interferon regulatory factor 3 (IRF3) in a myeloid differentiation primary response protein 88 (MyD88)–TIR-domain-containing adapter-inducing interferon-β-dependent pathway. Activated IRF3 then translocates to the nucleus and binds to target DNA sequences. Tumor necrosis factor (TNF) protein binds to its receptor and initiates inhibitor of κB (IκB)–NF-κB signaling pathway leading to translocation of NF-κB to the nucleus where it binds to AP-1 DNA sequences. Stimulation leukotriene B4 receptor 1 (LTB41) activates the c-Jun N-terminal kinase pathway, leading to phosphorylation and binding of the c-Jun–c-Fos heterodimer to target genes. NF-κB, c-Jun–c-Fos, and IRF3 induce expression of inflammatory factors such as cytokines, chemokines, and components of the inflammasome. When inflammasome is assembled, pro-caspase-1 is converted to caspase-1, which then converts pro-interleukin-1β (IL-1β) and pro-IL-18 to IL-1β and IL-18, respectively. I, insulin; insulin receptor; IRS, insulin receptor substrate.

The mammalian immune system consists of two types of immune responses: innate and adaptive. Innate immune cells include neutrophils, dendritic cells, macrophages, mast cells, and eosinophils, which respond to general danger signals associated with invading pathogens. Neutrophils are the first responders to invading pathogens and are generally among the first immune cells to arrive at the site of inflammation. Macrophages are long lived and highly dynamic. They readily switch from anti-inflammatory M2 type to pro-inflammatory M1-type in resident tissues. Besides bacterial danger signals mediated by lipopolysaccharide (LPS), the toll-like receptor 4 (TLR4) ligand, obesity-associated metabolic danger signals also play an important role in macrophage polarization. To provide local immune responses, macrophages get assistance from other immune cells, such as TLR-proficient mast cells (14). Eosinophils are anti-inflammatory in nature and maintain the M2 macrophage population. Adaptive immune cells include B-2 and T lymphocytes, which exert specific and decisive adaptive immune functions and provide immunological memory (15). B-2 and T lymphocytes are also involved in sterile inflammation and autoimmune disorders (16, 17). TNF-α released by M1 macrophage initiates inflammatory signaling through its receptor TNFR1 with consequent regulation of gene expression. In the cytoplasm, NF-κB is sequestered by the inhibitor of κB (IκB) to prevent nuclear translocation. The activation of the IκB kinase leads to phosphorylation of IκB and release of NF-κB, which then translocate to the nucleus and bind to the promoters of pro-inflammatory genes and initiates transcription (9, 18) (Figure 1). Alternatively, the inflammatory signaling can be initiated by the microbial-derived LPS, which acts through the TLRs. TLRs can sense lipids and saturated fatty acids and are able to induce activation of TLR2 and TLR4 through myeloid differentiation primary response protein 88-dependent pathways, whereas unsaturated fatty acids block TLR-mediated signaling pathways and gene expression (Figure 1). Receptors of advanced glycation end product bind to lipids and nucleic acids resulting in oxidative stress, activate NF-κB, and promote transcription of pro-inflammatory factors (19, 20) (Figure 1). The inflammasome, an oligomeric protein complex, comprises scaffold, adaptor, and caspase proteins that mediate the maturation and secretion of inflammatory cytokines interleukin-1β (IL-1β) and IL-18 (21). The NLR family pyrin domain containing 3 inflammasome recruits and activates pro-caspase 1 to produce caspase-1, which then cleaves pro-IL-1β and pro-IL-18 to mature IL-1β and IL-18, respectively (22).

## Immune Cells and Their Polarization in Adipose Tissue

**Figure 2:**
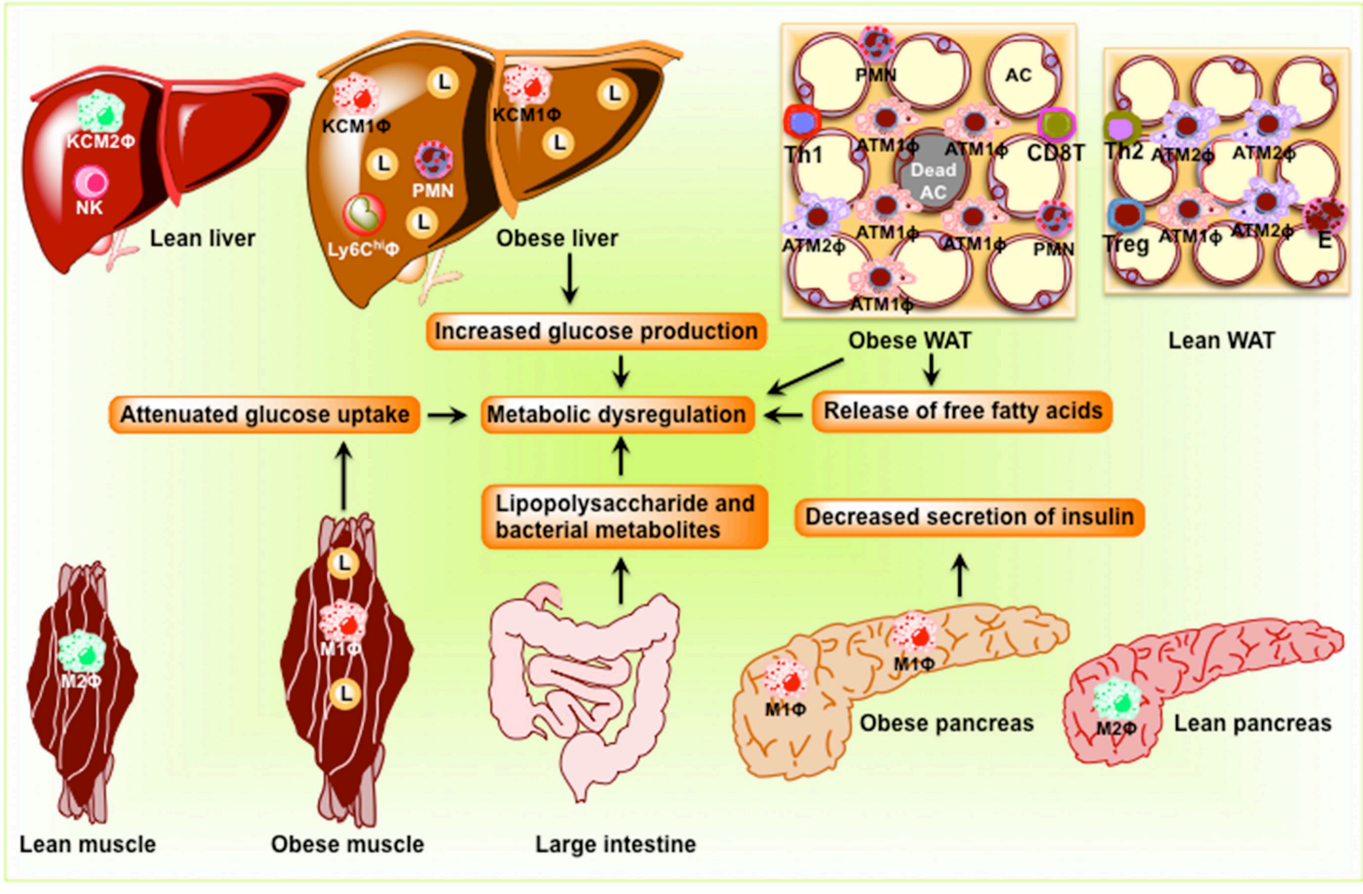
**Schematic diagram showing obesity-induced inflammation in peripheral organs including adipose tissue, the liver, skeletal muscle, and the pancreas to cause dysbiosis in the intestine**. In adipose tissue, pro-inflammatory signaling induces lipolysis and release of free fatty acids eventuating in the development of insulin resistance. In the liver, obesity induces pro-inflammatory cytokine production and M1 macrophage recruitment, resulting in insulin resistance and steatosis. In skeletal muscle of obese rodents, accumulations of lipid and pro-inflammatory macrophage inhibit insulin signaling, which result in the development of insulin resistance. In the pancreas, obesity induces macrophage infiltration, interleukin-1β secretion, and decreases insulin secretion. Because of the change in the composition of the microbial population, dysbiosis occurs in the intestine. AC, adipocyte; KC, Kupffer cell; L, lipid droplets; M1Φ, classically activated macrophages/pro-inflammatory macrophages; M2Φ, alternatively activated macrophages/anti-inflammatory macrophages; NK, natural killer cell; PMN, polymorphonuclear neutrophil; WAT, white adipose tissue.

The adipose tissue comprises adipocytes, immune cells (macrophages and lymphocytes), pre-adipocytes, and endothelial cells. Under lean conditions, Th2 T cells, T_reg_ cells, eosinophils, and ATM2-like resident macrophages predominate in the adipose tissue (Figure 2). ATM2 macrophages express CD11b, F4/80, CD301, and CD206 and promote local insulin sensitivity through production of anti-inflammatory cytokines, such as IL-10 (18). T_reg_ cells not only secrete IL-10 but also stimulate ATM2 macrophage to secrete IL-10. Eosinophils, on the other hand, secrete IL-4 and IL-13. In the lean state, IL-4, IL-10, and IL-13 maintain the anti-inflammatory and insulin-sensitive phenotype. In contrast, obesity induces lipolysis and release of pro-inflammatory free fatty acids (FFAs) and factors such as C–C motif ligand 2 (CCL2) and TNF-α that recruit blood monocytes in adipose tissue, where they become polarized to the highly pro-inflammatory M1-like state (Figure 2). FFAs serve as ligands for the TLR4 complex (23), activate classical inflammatory response, and drive accumulation of ATM (24, 25). Activated ATM1 express CD11c in addition to CD11b and F4/80 and produce large amounts of pro-inflammatory mediators such as TNF-α, IL-1β, IL-6, leukotriene B4, NO, and resistin that work in a paracrine fashion and causes IR in adipose tissue (26). The anti-inflammatory eosinophil population declines in obese adipose tissue. In addition, obesity decreases T_reg_ content and an increase in CD4^+^ Th1 and CD8^+^ effector T cells, which also secrete pro-inflammatory cytokines. Obesity increases B cell numbers and activates T cells, which potentiate M1-like macrophage polarization, inflammation, and IR. Cytokines and chemokines are also released from the adipose tissue and promote inflammation and consequent IR in liver, muscle, and pancreas.

## Immune Cells and Their Polarization in Liver

In the lean liver, hepatocytes are the major parenchymal cells, while the non-parenchymal cells integrate five cell populations including resident macrophages of M2-type or Kupffer cells (27), recruited hepatic macrophages, resident innate lymphocytes or natural killer cells (NKs) (28, 29), fat storing cells termed Ito or stellate cells (HSCs) (30), and liver sinusoidal endothelial cells (LSECs) (31). Under lean conditions, Kupffer cells (KCs) in collaboration with other hepatic immune cell populations clear microbial material while maintaining the inflammatory tone of the liver at a level sufficient for essential functions such as pathogen killing, tissue remodeling, and sinusoidal permeability, but below that they would result in overt inflammation and tissue damage (32–34). NKs eliminate virus-infected or transformed cells and regulate adaptive immune responses *via* contact-dependent signals and the secretion of cytokines (35–38).

Hepatic lipid accumulation and peroxidation lead to chronic hepatocyte endoplasmic reticulum stress, the production of reactive oxygen species, and TLR activation, which converts KCs into an M1 phenotype defined by production of pro-inflammatory cytokines, oncostatin, and prostaglandins (PGE_2_) (39–41). Circulating cytokines, adipokines, and FFAs released from inflamed adipose tissue in the obese state or immunogenic material derived from an altered intestinal microbiota can also contribute to KC polarization. M1-KCs secrete chemokine CCL2 (also known as MCP1), pro-inflammatory cytokines (TNF-α, IL-1β, and IL-6), macrophage inflammatory protein (MIP)-1a, MIP1b, RANTES, oncostatin, and PGE_2_, which contribute to the alteration of the liver homeostasis and worsen the hepatic inflammatory response (42). PGE_2_ regulates cytokine production (IL-1β, IL-6, TNF-α, and TGF-β) (43, 44), acts synergistically with IL-6 to induce IR (45), and induces production of oncostatin M (OSM) in KCs (46). Increased OSM contributes to hepatic IR and the development of NASH (46). High levels of TNF-α released by M1-KCs stimulates hepatic expression of CCL2, a powerful monocyte chemoattractant, which recruits CCR2^+^Ly6C^high^ monocytes from the vasculature into the liver (47), where they differentiate into Ly6C^high^ macrophages. The Ly6C^high^ macrophages amplify the severity of obesity-induced inflammation and hepatic IR through secretion of TNF-α and IL-6 (48).

## Adipose Tissue Fibrosis and Metabolic Dysfunction

Adipocytes and their progenitor cells (pre-adipocytes) are embedded in a network of extracellular matrix (ECM), which tightly regulates the function of adipose tissue (49). Fibrosis, the excessive accumulation of ECM components, is a highly conserved and coordinated protective response to tissue injury and is a common pathological consequence of inflammatory diseases (50). Fibrosis develops from an imbalance between excess synthesis of ECM components including collagens (I, III, and VI), elastins, and proteoglycans (51, 52), and an impairment in degradation of these proteins. Fibrosis limits the expandability of adipose tissue and contributes to ectopic fat accumulation and the development of IR (53). It has been recently shown that treatment with the antidiabetic drug metformin inhibits excessive ECM deposition in white adipose tissue (WAT) of leptin-deficient *ob/ob* mice and mice with diet-induced obesity (54). Fibrotic disorders cause 45% deaths in the United States (52). In adipose tissues, ECM undergoes constant remodeling to allow adipocytes to rapidly expand and shrink in parallel with weight gain and loss and function in adaptation to nutritional clues (55). Adipocytes undergo dramatic expansion during the development of obesity. Macrophages are believed to be the master “regulators” of fibrosis as they produce soluble mediators including TGF-β1 and platelet-derived growth factor (PDGF), which directly activate fibroblasts and control ECM dynamics by regulating the balance of various matrix metalloproteinases (MMPs) and tissue inhibitors of MMP (TIMP) (56). Myofibroblasts, macrophages, and endothelial cells also produce MMP and TIMP for ECM regulation (57). While MMPs are responsible for the degradation of virtually all ECM proteins (58), TIMP inhibits MMPs and is responsible for degrading excess ECM (59). Macrophages also regulate fibrogenesis by releasing chemokines and attract fibroblasts and other inflammatory cells. Thus, IL-13 produced by Th2 CD4^+^ T cells (52, 60, 61) and TGF-β1 activate fibroblasts to differentiate into α-smooth muscle actin (α-SMA) expressing myofibroblasts to produce ECM (62–64).

## Liver Fibrosis and Metabolic Dysfunction

Liver fibrosis results from the would-healing response of the liver to repeated injury such as hepatitis C virus (HCV) infection, alcohol abuse, and NASH (65, 66). Fibrosis is increasingly appreciated as a major contributor to metabolic dysregulation in obese humans and T2D patients (67). Advanced liver fibrosis leads to cirrhosis and death (68). Increased gut permeability and hepatic TLR4 signaling promotes fibrogenesis. Both KCs and recruited Ly6C^high^ macrophages contribute to the development of hepatic fibrosis (69). HSCs are the main collagen-producing cells in liver (70, 71). KCs activate HSCs through increased production of profibrotic cytokine TGF-β and mitogenic PDGF (72) leading to fibrosis. TGF-β leads to transdifferentiation of HSCs into myofibroblasts. PDGF stimulates myofibroblast proliferation. Inhibition of PDGF by anti-sense strategy attenuates liver fibrogenesis (73). HSC-derived myofibroblasts express α-SMA and collagen I. During fibrogenesis, LY6C^high^ monocytes are recruited to the inflamed liver *via* the CCL2/CCR2 (C–C chemokine receptor type 2) axis, forming a profibrotic Ly6C^high^ macrophage, which has been shown to be the predominant pro-fibrogenic population in the liver (74, 75). These cells express TNF-α and IL-1β, which perpetuate hepatocellular injury and enhance the survival of hepatic myofibroblasts. In addition, Ly6C^high^ macrophages express high levels of TGF-β-activating thrombospondin 1 (76). Macrophages also express the potent mitogen PDGF and the Th2 cell cytokines IL-4 and IL-13, which directly stimulate collagen synthesis in myofibroblasts. Chemokine expression such as CCL8 (also known as MCP2) and CCL7 (also known as MCP3) by these macrophages promotes the recruitment of monocytes, other inflammatory cells, and HSCs (77). Ly6C^high^ macrophages also interact with HSCs to promote fibrosis through increased production of TGF-β, connective tissue growth factor (CTGF), and PDGF (78). Inhibition of the main monocyte chemoattractant CCL2 in rats or genetic deletion of its receptor CCR2 in mice decreased macrophage infiltration in response to injury and markedly inhibited liver fibrosis, implicating monocyte recruitment as an essential component in liver fibrogenesis (78–82). In addition, pharmacological inhibition of CCL2 by the RNA-aptamer mNOX-E36 attenuates liver fibrosis, thereby strengthening a profibrotic function of Ly6C^high^ macrophages (83, 84). Hepatic myofibroblasts express TIMP1, which inhibits MMP activity and augments the accumulation of ECM in the scar tissue.

## Obesity, Tissue Inflammation, and Insulin Resistance

Components of the immune system are affected in obesity and T2D and inflammation participates in the pathogenesis of T2D. Thus, obesity affects the immune system and promotes inflammation with consequent development of IR (85–87). Obesity-induced increased levels of glucose and FFAs create stress in pancreatic islets, adipose tissue, liver, and muscle, resulting in increased local production and release of cytokines and chemokines such as IL-1β, TNFα, CCL2, CCL3, and CXC-chemokine ligand 8 (CXCL8, also known as IL-8). These changes promote recruitment of immune cells in insulin-sensitive tissues and contribute to tissue inflammation and further production and release of cytokines and chemokines. The augmented release of cytokines and chemokines promotes inflammation in liver, muscle, and pancreatic islets. Obesity affects insulin signaling and causes IR by the following mechanisms: (i) inflammatory stimuli phosphorylate IκB resulting its dissociation from IκB/NF-κB complex followed by degradation in the cytoplasm. This allows translocation of free NF-κB to the nucleus, where it binds to cognate DNA response elements and transactivates the transcription of inflammatory genes. (ii) Phosphorylation and activation of c-Jun N-terminal kinase (JNK) leading to phosphorylation of the N-terminus of c-Jun. This initiates a switch of c-Jun dimers for c-Jun–c-Fos heterodimers with consequent stimulation of transcription of inflammatory target genes. (iii) Production of “second messengers,” such as FFAs, that promote IR. (iv) Augmented transcription of genes involved in lipid processing, including the enzymes that synthesize ceramide, which inhibits the activation of AKT (88, 89).

Recent studies in both rodents and humans implicate gut microbiota as a contributor to metabolic disorders (90). The gut microbiota plays a part in the host’s genomic profile and metabolic efficiency (91). Obesity in humans and rodents is associated with changes in the composition of the intestinal microbiota (92, 93). Dysbiotic microbiota in obesity enhances the digestion of complex carbohydrates and macronutrient absorption, leading to the development of obesity (94). In addition, gut microbiota has the capacity to harvest energy from nutrients and stores energy in the form of fat (95). The gut microbiota is also capable of inducing “metabolic endotoxemia” by increasing exposure to bacterial LPS coming from gut (96). LPS in the bloodstream contributes to IR by promoting tissue inflammation (97, 98).

## Obesity and NAFLDs

Non-alcoholic fatty liver disease, the liver manifestation of the metabolic syndrome, has become the most common disorder in the United States and other developed countries, affecting over a third of the population (99). NAFLD begins with a simple steatosis that may evolve into NASH, a medley of inflammation, hepatocellular injury, and fibrosis, often resulting in cirrhosis and even hepatocellular cancer (100–102). KCs differ in their population density, morphological characteristics, and physiological functions depending on their position within the liver sinusoids (103, 104). Severity of human NAFLD is associated with higher population of KCs (105). However, NASH is associated with aggregates of enlarged KCs (106). Selective depletion of large KCs by administration of gadolinium chloride markedly attenuates liver injury induced by thioacetamide (107), carbon tetrachloride (108), alcohol (109), and ischemia/reperfusion (110), indicating the critical roles played by larger KCs in liver damage in these condition. In experimental NAFLD induced by methionine/choline deficient diet, liposome-encapsulated dichloromethylene bisphosphonate (clodronate) effective blunts all histological evidence of NASH (111). These findings indicate that the activation of KCs positioned at the “frontline” is an essential element in the pathogenesis of NAFLD similar to other types of liver injury.

## Therapeutic Perspectives on Immunomodulation

Although it is yet to be definitely established whether tissue inflammation causes IR in humans, several anti-inflammatory approaches have been tested in clinical studies of obese individuals with IR. Thus, salsalate, an analog of salicylate, has been shown to improve insulin clearance and insulin sensitivity (112–115). Anti-TNF antibodies were found to decrease blood glucose in obese individuals (116). Anti-IL-1β monoclonal antibody therapy improved glycemic condition and β-cell insulin secretion (117–119). The antidiabetic thiazolidinediones (e.g., rosiglitazone and pioglitazone) decreased adipose tissue macrophage content (120, 121) and increase circulating levels of adiponectin and FGF21, thereby mediating redistribution of adipose tissue lipid stores (122, 123). Orexin-1 receptor antagonist has been shown to exert anti-obesity effects in obese leptin-deficient *ob/ob* mice (124, 125). While obese mice fed a high-fat diet supplemented with ω-3 fatty acids caused a decrease in inflammation, improved insulin sensitivity, and normalized glucose tolerance (126), fish-oil supplementation yielded mixed results on metabolic end points in human studies (127, 128).

## Conclusion and Future Perspectives

Although the last 15 years has witnessed a renaissance in the field of immunology and metabolism, immunometabolism is still a young field with many questions to be answered. (i) To what extent are obesity and inflammation triggered in parallel or in sequence? (ii) What is the ontogeny and fate of stromal cells that populate WAT and liver? (iii) Do macrophage localization and origin regulate immunometabolic phenotype? (iv) By what pathway(s) does inflammation provoke T2D? (v) Can genetic and environmental factors reinforce or dissociate the link between metabolic and immunological abnormalities? (vi) Do anti-inflammatory strategies target the underlying mechanisms of the disease, and if so, would starting these therapies early prevent progression or even the overt manifestation of the disease? Answers to the above questions and a more detailed understanding of immunometabolism will permit more focused immune therapies to target metabolic diseases.

## Author Contributions

IR and RD researched data and wrote the first draft of the article. SM researched data and extensively revised the draft, and made both the figures.

## Conflict of Interest Statement

The authors declare that the research was conducted in the absence of any commercial or financial relationships that could be construed as a potential conflict of interest.
